# ZAP’s stress granule localization is correlated with its antiviral activity and induced by virus replication

**DOI:** 10.1371/journal.ppat.1007798

**Published:** 2019-05-22

**Authors:** Lok Man John Law, Brandon S. Razooky, Melody M. H. Li, Shihyun You, Andrea Jurado, Charles M. Rice, Margaret R. MacDonald

**Affiliations:** The Laboratory of Virology and Infectious Disease, The Rockefeller University, New York, NY, United States of America; The University of Chicago, UNITED STATES

## Abstract

Cellular antiviral programs encode molecules capable of targeting multiple steps in the virus lifecycle. Zinc-finger antiviral protein (ZAP) is a central and general regulator of antiviral activity that targets pathogen mRNA stability and translation. ZAP is diffusely cytoplasmic, but upon infection ZAP is targeted to particular cytoplasmic structures, termed stress granules (SGs). However, it remains unclear if ZAP’s antiviral activity correlates with SG localization, and what molecular cues are required to induce this localization event. Here, we use Sindbis virus (SINV) as a model infection and find that ZAP’s localization to SGs can be transient. Sometimes no apparent viral infection follows ZAP SG localization but ZAP SG localization always precedes accumulation of SINV non-structural protein, suggesting virus replication processes trigger SG formation and ZAP recruitment. Data from single-molecule RNA FISH corroborates this finding as the majority of cells with ZAP localization in SGs contain low levels of viral RNA. Furthermore, ZAP recruitment to SGs occurred in ZAP-expressing cells when co-cultured with cells replicating full-length SINV, but not when co-cultured with cells replicating a SINV replicon. ZAP recruitment to SGs is functionally important as a panel of alanine ZAP mutants indicate that the anti-SINV activity is correlated with ZAP’s ability to localize to SGs. As ZAP is a central component of the cellular antiviral programs, these data provide further evidence that SGs are an important cytoplasmic antiviral hub. These findings provide insight into how antiviral components are regulated upon virus infection to inhibit virus spread.

## Author summary

Organisms encode immune programs, present in most somatic cells, to combat pathogens. The components of these antiviral programs are both constitutively expressed and highly upregulated upon pathogen recognition. Interestingly, a broadly acting antiviral factor is the zinc-finger antiviral protein (ZAP). ZAP is a primarily cytoplasmic protein that upon various cellular stresses, such as virus infection, can localize to specific cytoplasmic complexes termed stress granules (SGs). SGs are hubs that regulate mRNA stability and translation. Here, we show that SG localization is (*i*) correlated with ZAP’s antiviral function, (*ii*) most likely triggered during the early stages of virus replication, and (*iii*) a highly dynamic and transient process. Collectively, our data highlight the genetic and dynamic components of ZAP-mediated antiviral activity.

## Introduction

Innate immunity is comprised of a set of cellular factors whose function is to act as the ‘front-line’ defense against invading pathogens [1,2]. Some innate components are constitutively expressed, allowing for constant surveillance and inhibition of pathogens, while others are activated upon pathogen sensing or by the interferon cytokines [3]. Upon pathogen recognition, the interferon cytokines are secreted and sensed by cells, leading to the induction of interferon stimulated genes (ISGs) that act to recognize and halt pathogen replication and spread. One classic example of an innate immunity component is the zinc-finger antiviral protein (ZAP), which is constitutively expressed in many cells and induced upon pathogen sensing and in response to interferon [4–6]. Initially identified as an anti-Moloney murine leukemia virus (MLV) factor [7], ZAP exhibits broad antiviral activity, for example inhibiting other retroviruses [8], LINE-1 retrotransposition [9], alphaviruses [10], filoviruses [11], enteroviruses [12], hepatitis B virus [13], influenza [14] and porcine reproductive and respiratory virus [15]. While it is not clear how ZAP is able to exert such broad antiviral function, a recent study postulated that increased CpG content may underlie susceptibility of a virus to ZAP’s antiviral effects [16].

ZAP is encoded by the zinc finger CCCH-type antiviral 1 (*ZC3HAV1*) gene, and two alternative splice variants have been identified that differ by the presence of a catalytically inactive poly (ADP-ribose) polymerase (PARP) domain in the longer isoform. Because of the presence of this domain, ZAP is also known as PARP13 (for review see [17]). Both isoforms have antiviral function as they each contain the minimal antiviral domain located within the amino-terminal third of the proteins [7,18], although the longer isoform has been reported to have higher antiviral activity [19]. Within the antiviral amino-terminal end, there are four CCCH type zinc fingers that mediate RNA binding activity [20]. These CCCH domains bind to RNA responsive elements in viruses, promoting RNA degradation via interaction with host helicase and exosome components [21–23]. For some viruses, such as SINV and MLV, the virus regions where ZAP binds were mapped [24]. Mechanistically, ZAP targeting of the viral genome, at least for SINV, is thought to block RNA replication by binding to and inhibiting the translation of the incoming genome [10]. Inhibition, knockout or knockdown of endogenous ZAP allows for higher levels of SINV replication in cell culture and murine models, demonstrating that endogenous ZAP levels can mediate antiviral effects [18,25–28].

SINV infection is an ideal model system to study ZAP’s antiviral activity as SINV has been extensively studied as the prototype of alphaviruses (for review see [29]). The genome of SINV is a positive-strand RNA molecule encoding two open reading frames (ORFs). The 5’ proximal ORF is translated into a polyprotein that gets processed into nonstructural proteins (nsPs) 1–4, which are responsible for the replication of the SINV genome at evaginations of the plasma membrane [30,31]. Structural proteins are translated from a subgenomic RNA (3’ proximal ORF) and package the viral RNA to form new virions [32,33]. Like many other viruses, SINV must co-opt host-cell translation machinery despite the activation of inhibitory pathways during infection. Specifically, cells reduce global cap-dependent translation through PKR-mediated phosphorylation of elF2α during SINV infection [34], but SINV is capable of sustaining translation of viral RNA via an eIF2-independent translational element [34,35]. Interestingly, ZAP seems to play a central role in antagonizing SINV as ZAP overexpression renders SINV sensitive to translation inhibition [10].

Upon virus infection, as well as other stress conditions, ZAP localizes to punctae in the cytoplasm termed stress granules (SGs), which play an important role in regulating cellular translation [31,32]. Stalled translation complexes get recruited to SGs during cellular stress in order to prioritize the translation of a subset of stress-related genes. SGs are an important hub to initiate antiviral processes; two well studied virus sensing pattern recognition receptors, retinoic acid-inducible gene I (RIG-I) and melanoma differentiation-associated gene 5 (MDA5), also localize to SGs upon infection [36,37]. Despite the numerous antiviral proteins present in SGs, their role in infection remains unclear. In some instances, viruses actively prevent SG formation to permit efficient replication by cleaving SG components [38], while in others, proteins normally associated with SGs can also be required for viral replication. During flavivirus infection, the virus co-opts the SG-component eIF4E, a translation initiation factor, in order to favor translation of viral transcripts [39]. During SINV infection, the viral nsP3 protein has been shown to interact with another SG localized protein, G3BP, and this interaction is important for its replication [30,40,41]. Interestingly, G3BP is a common virus target for co-option of SG function [42,43]. G3BP has also been implicated, through its interaction with PKR, to potentiate the innate immune response [44]. While it is clear that G3BP actively participates in the replication of SINV [45], it is not known whether SINV interaction with G3BP dampens PKR or SG mediated innate immune signaling. Infection with Semliki Forest virus (SFV), another alphavirus, has been shown to cause the formation and then dissolution of SGs [46]. Thus, viruses can overcome translation repression by either inhibiting SG formation or co-opting SG function.

Though ZAP, along with other members of the PARP family, has been shown to localize to SG structures [47], the importance of SG targeting by ZAP is poorly defined. One hypothesis is that ZAP interacts with Ago2 in SGs to relieve miRNA mediated translation repression [47]. Here, based on previous findings that ZAP affects SINV translation and that ZAP is targeted to SGs, we investigated the involvement of SGs in ZAP’s anti-SINV activity. We map the features of ZAP important for targeting to SGs and relate these features to ZAP’s antiviral activity. Using time-lapse imaging, we also observe the dynamics of ZAP localization to SGs in response to SINV infection and how this localization affects SINV infection. By coupling single-molecule RNA fluorescence *in situ* hybridization (smFISH) with time-lapse microscopy, our data suggests that ZAP localization during the early stages of infection acts to inhibit virus replication, corroborating a previous hypothesis that ZAP stalls the translation of SINV RNA in order to prevent productive infection.

## Results

### ZAP attenuates SINV replication and localizes to form concentrated punctae within the cytosol of U2OS cells

**Fig 1:**
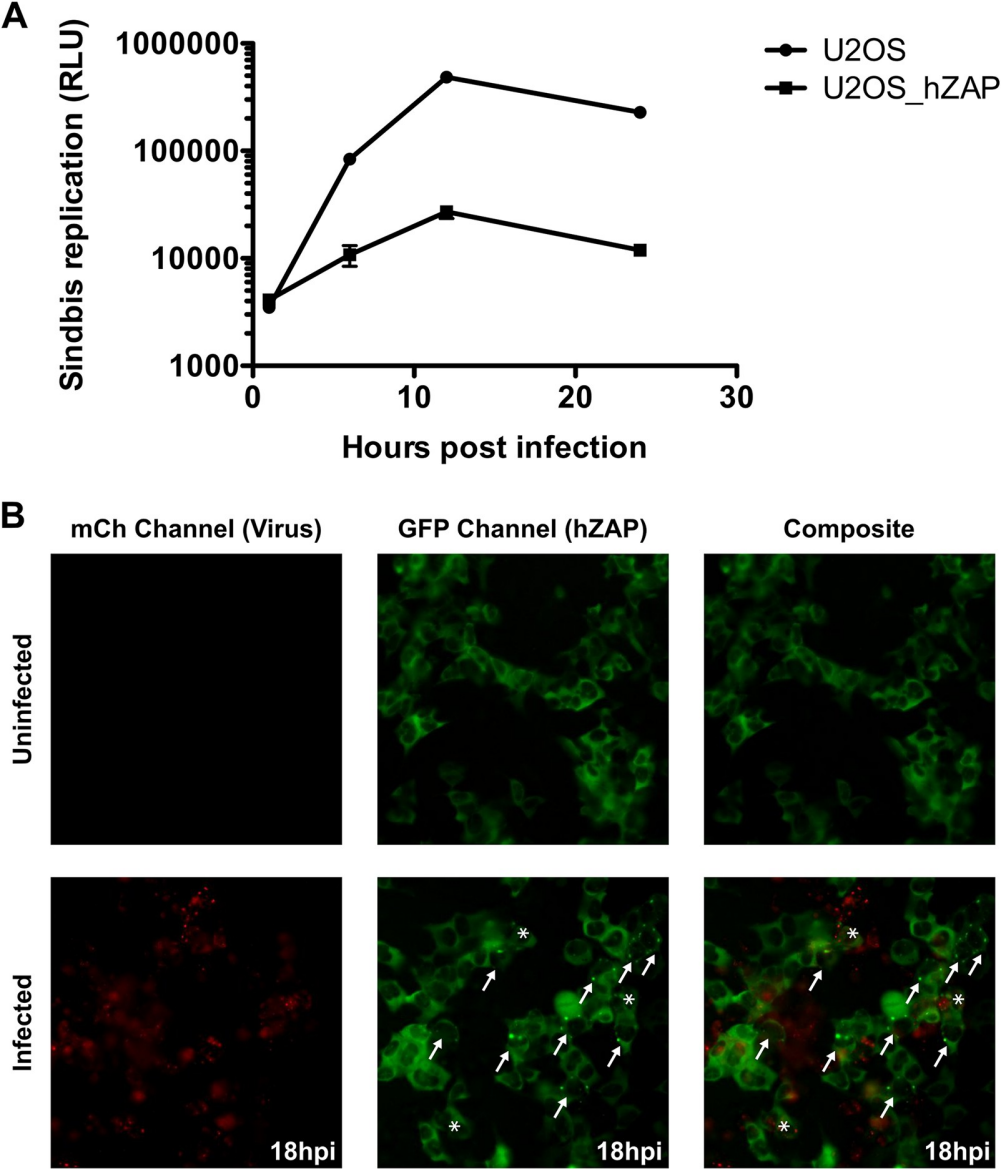
ZAP inhibits SINV replication and can relocate to clusters in the cytosol upon infection. (A) U2OS cells ectopically expressing GFP-tagged hZAP (U2OS_hZAP, squares) or GFP as control (U2OS, circles) were infected with luciferase-expressing SINV at an MOI of 10. At the indicated times cells were lysed and luciferase activity was measured. RLU, relative light units. Error bars represent standard error of the mean from triplicate samples and are often obscured by the symbol. (B) A co-culture of naïve U2OS cells and U2OS cells ectopically expressing hZAP-GFP (4:1 ratio) were mock infected (top row) or infected with SINV Toto1101 expressing an nsP3-mCherry (MOI = 1 (bottom row)). Cells were visualized at 18hpi for nsP3-mCherry and hZAP-GFP on a fluorescent microscope and the individual and merged (composite) images are shown. Arrows point to cells containing hZAP-GFP punctae within the cytosol and asterisks indicate infected hZAP-GFP cells.

In this study, we aimed to examine in more detail the relationship between the anti-SINV activity of ZAP and SG formation. In order to achieve optimal images, we utilized U2OS cells, which are human bone osteosarcoma epithelial cells that have a flat shape and large cytoplasm, which is ideal for imaging cellular structures. We first validated the ability of ZAP to act as an anti-SINV factor in this cell line. Fig 1A shows that overexpression of ZAP reduced SINV replication by more than 10-fold. Exposure of U2OS cells to SINV caused ZAP, which is known to shuttle between the nucleus and cytoplasm [48], to shift from its diffuse cytoplasmic staining to discrete puncta (Fig 1B, arrows). In some instances, we observed double positive cells indicative of infection in cells with ectopic ZAP expression (Fig 1B and S1 Movie).

### ZAP is targeted to SGs, not P-bodies, during stress

**Fig 2:**
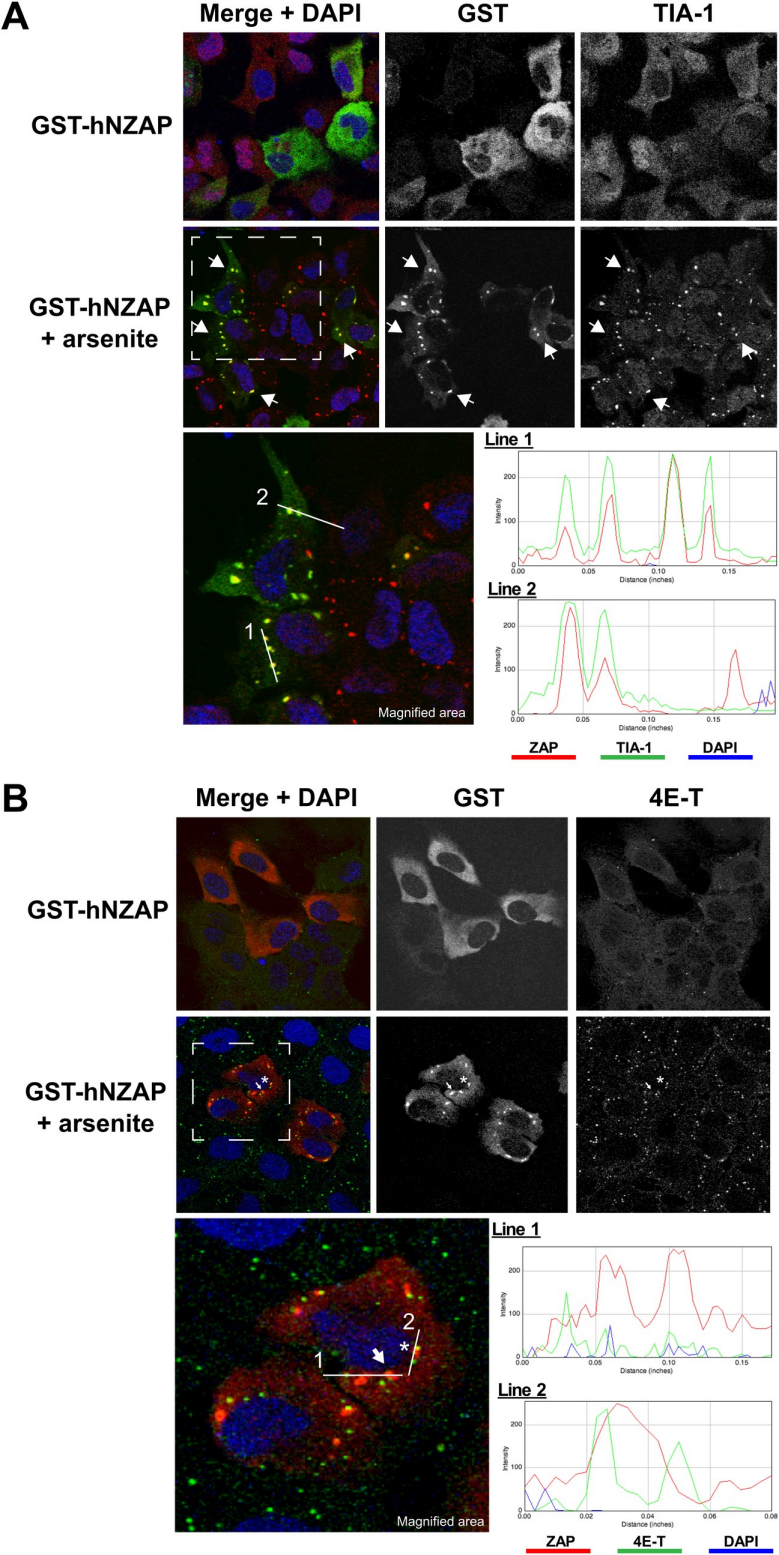
ZAP is targeted to SGs, but not P-bodies, in response to stress. (A) U2OS cells transiently expressing GST-hNZAP were treated with or without 0.5 mM arsenite for 30 minutes. Localization of GST tagged ZAP was monitored by immunofluorescence using an anti-GST antibody, while SGs were visualized using antibodies to the stress granule marker TIA-1 and examined by confocal microscopy. Examples of colocalization between ZAP and TIA-1 are highlighted by arrows. The area enclosed by the white dashed line is magnified and shown below. After oxidative stress, ZAP localized to foci positive for TIA-1 (arrows). Histograms along line 1 and 2 of each color profile are shown to illustrate colocalization of ZAP and TIA-1. (B) After response to arsenite stress cells were also stained with the host P-body marker 4E-T and examined by confocal microscopy. An example of colocalization between ZAP and 4E-T is highlighted with an asterisk (*) and ZAP not localized with 4E-T is marked with an arrow. The area enclosed by the white dashed line is magnified and shown below. Histograms of each color profile are shown to demonstrate ZAP that is not (line 1) or is (line 2) associated with P-bodies after oxidative stress.

ZAP has been shown to be targeted to SGs under certain conditions [47]. In order to understand the SG localization and ZAP’s anti-SINV activity, we first investigated if the amino-terminal third of the protein, designated NZAP and containing the minimal antiviral domain of ZAP (amino acids 1–252) [18], is sufficient for SG targeting. Overexpressed GST-tagged NZAP showed a diffuse cytoplasmic distribution (Fig 2A, top panels). Upon exposure to oxidative stress mediated by treatment with arsenite, NZAP localized to a more punctate staining overlapping with the SG marker TIA-1 (Fig 2A, bottom panels) [49]. These results demonstrate that, like ZAP, NZAP is actively targeted to SGs in response to cellular stress and suggest that the SG-targeting residues of ZAP reside within the minimal antiviral domain of ZAP.

In some cases, SGs are found in close proximity to another type of RNA granule known as P-bodies, which function in mRNA decay (reviewed in [50]). The functions of SGs and P-bodies are closely linked, but distinct [51]. Without induction of the stress response, staining with antibody to the P-body resident protein 4E-T showed a small number of P-bodies in each cell (Fig 2B, top panels). Upon arsenite treatment, we observed that NZAP localized to SGs and the number of P-bodies increased (Fig 2B, bottom panels). A fraction of the NZAP-containing SGs were in close proximity to P-bodies and in some cases overlapped with P bodies; however, the majority of NZAP-containing SGs did not colocalize with P-bodies. Based on the localization of NZAP to SGs during stress, the ZAP-containing punctae seen upon exposure of the cultured cells to SINV (Fig 1B) were likely SGs.

### ZAP localizes to SGs during SINV infection

**Fig 3:**
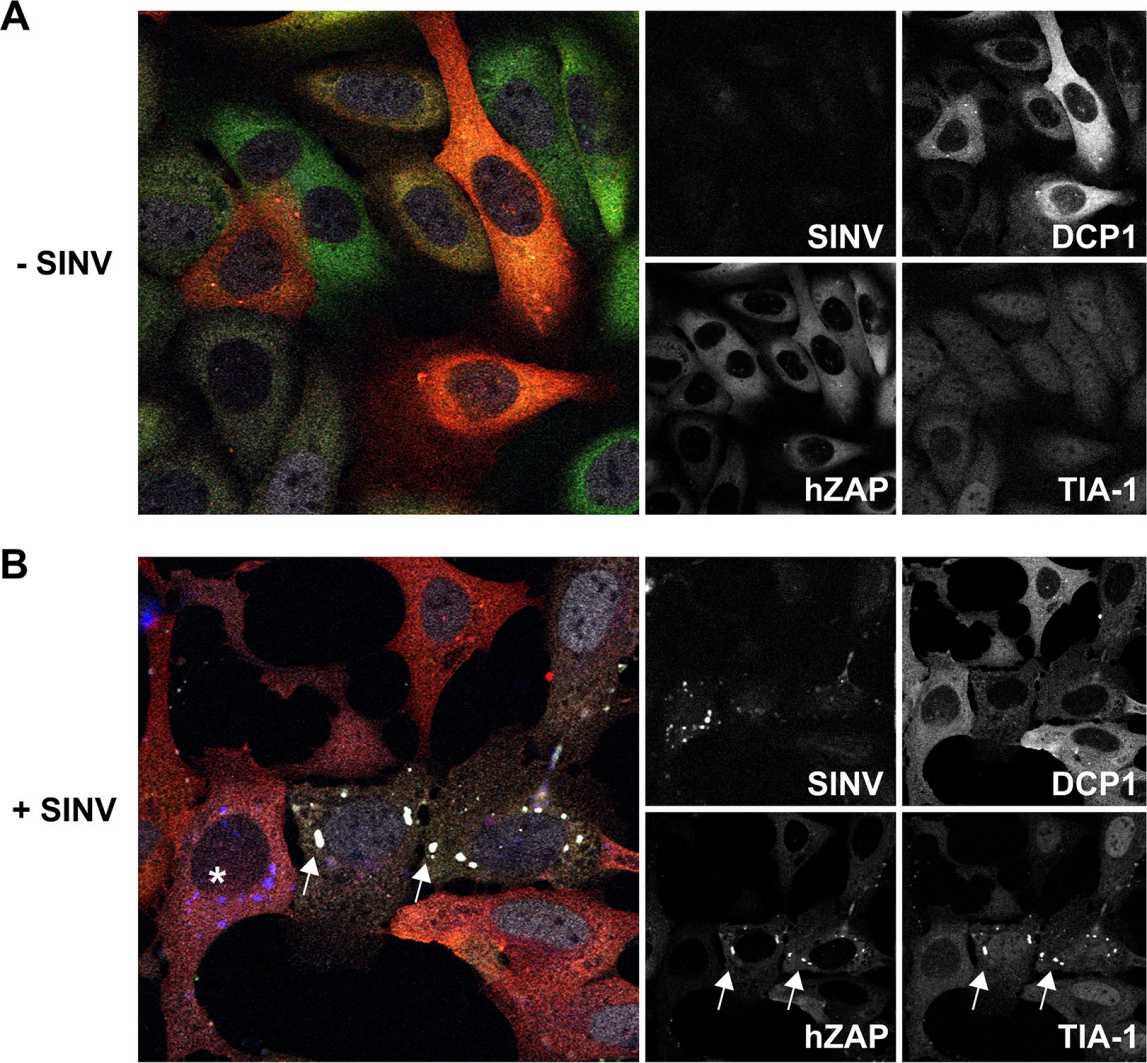
SINV infection causes localization of ZAP to granules containing TIA-1, but not DCP1. (A, B) Heterogeneous populations of U2OS cells transduced to express hZAP-GFP and RFP-DCP1 (P-body marker) were left uninfected (A) or were infected (B) with BFP-tagged SINV at MOI = 1. At 20 hpi, cells were fixed and stained with anti-TIA-1 antibodies to mark SGs followed by Alexafluor 594 secondary antibody. Individual images are shown to the right, and the merged images (left) were pseudo-colored: Blue (BFP-Sindbis); Green (GFP-ZAP); Red (RFP-DCP1) and Grey (TIA-1). An infected cell is marked with an asterisk and arrows show colocalization of hZAP and TIA-1.

To confirm that the foci to which ZAP localizes in the context of infection are indeed SGs, we utilized a cell line overexpressing GFP-tagged hZAP and ectopically expressed RFP-tagged DCP1, a P-body cellular marker, and used indirect immunofluorescence to localize the SG marker TIA-1. Expression of ZAP and DCP1 was heterogeneous, as some cells overexpressed ZAP, some DCP1, and some both proteins (Fig 3A). In uninfected cells, overexpressed ZAP was found diffusely in the cytoplasm. We infected these cells with recombinant SINV expressing BFP-tagged nsP3 (nsP3-BFP) (Fig 3B). Interestingly, in cells adjacent to the SINV-infected cells with high levels of nsP3-BFP expression, ZAP localized to punctae similar to Fig 1B (Fig 3B). These punctae contained the SG marker TIA-1 without a change in DCP1 localization, suggesting that the ZAP-containing punctae seen after SINV infection are SGs, but not P-bodies. Interestingly, the punctate localization of ZAP occurred in cells without or with low levels of nsP3-BFP expression, whereas ZAP and TIA-1 remained diffuse in cultures not exposed to SINV (Fig 3A). The data suggests that targeting of ZAP to SGs may either require direct SINV infection, a signal from neighboring infected cells, or both.

### Formation of ZAP-containing SGs does not always lead to noticeable accumulation of viral proteins, but actively infected cells always form SGs

**Fig 4:**
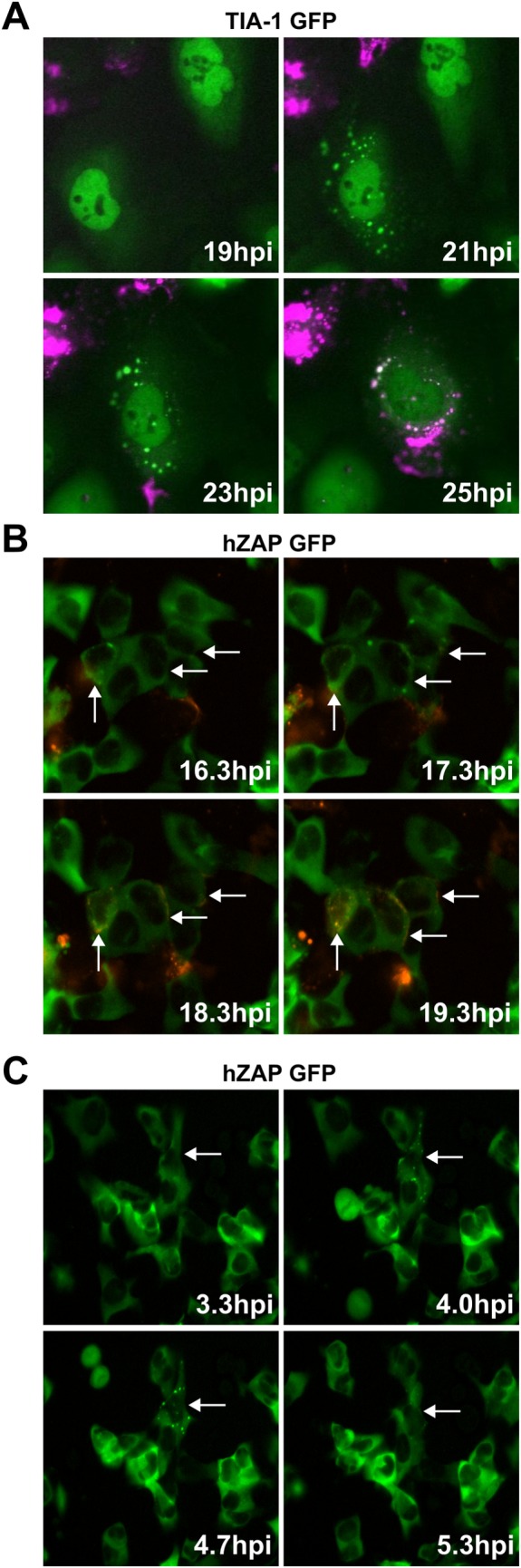
hZAP localization occurs in every infected cell but many cells showing ZAP localization do not show signs of virus replication. (A) U2OS cells stably expressing GFP-tagged TIA-1 (green) were infected with SINV encoding nsP3-mCherry (magneta). The effect of SINV infection on SG was monitored by time-lapse imaging. Widefield fluorescent images were taken every 20 minutes between 14–48 hpi and the timing of SG formation relative to SINV infection (nsP3-mCherry expression) was monitored in multiple fields. A montage of selected time points of a representative field is shown (about 19 to 25 hpi). (B and C) Naïve U2OS cells and U2OS cells expressing hZAP-GFP mixed at a 4:1 ratio were infected with SINV expressing mCherry-tagged nsP3. Time-lapse microscopy of GFP and mCherry expression was performed with an image taken every 20 minutes for 24 hrs to assess ZAP localization and evidence of infection. (B) Images from a representative field taken at a later time point. Cells that show initial ZAP localization and subsequent nsP3-mCherry expression are indicated with arrows. (C) Images from a representative field taken at an early time point. Arrows point to a cell with SG formation during this window of time.

To properly understand the dynamics of SG formation in the context of endogenous ZAP levels during SINV infection and to understand the ‘fate’ of each infected cell, we quantified the incidence of infection in cells overexpressing GFP-tagged TIA-1 and showing SG localization (Fig 4A) using fluorescence time-lapse microscopy. Cells were imaged and GFP-tagged TIA-1 and mCherry-tagged nsP3 (nsP3-mCherry) of SINV were followed over time in single-cells. The majority of cells that showed TIA-1 localization into SG punctae (Fig 4A) were infected, (15/19 = 79%) as demonstrated by accumulation of nsP3-mCherry. The formation of SGs, as assessed by TIA-1 localization, preceded visible accumulation of nsP3-mCherry (Fig 4A and S1 Appendix and S1 Movie). Thus, early SINV replication processes most likely trigger SG formation.

To further characterize ZAP localization to SGs during SINV infection, we used time-lapse imaging of infected hZAP-GFP expressing cells. U2OS cells overexpressing hZAP-GFP were infected with SINV encoding nsP3-mCherry and monitored from 0 hours post-infection (hpi) to 24 hpi (Fig 4B and 4C and S1 Movie). Naïve U2OS cells were mixed at a 4:1 ratio to U2OS hZAP-GFP cells as hZAP ectopic expression renders cells more refractory to virus infection. Co-culture conditions allow for virus spread and an increased frequency of hZAP-GFP cell exposure to SINV. In mock-infected cells, ZAP did not show relocation to SGs and instead showed cytoplasmic staining throughout the imaging period (Fig 1). However, in the cultures exposed to SINV, as seen previously (Figs 1B and 3B), ZAP was targeted to SGs during SINV infection. Interestingly, ZAP targeting to SGs was sometimes transient as ZAP localization to these structures lasted around 1–2 hours before dissolving (Fig 4C and S1 Movie).

To test if ZAP recruitment to SGs is correlated with SINV infection, the entire imaging period, 24 hours, was quantified by first checking in the hZAP-GFP-expressing cells whether there was evidence for infection (double positive for mCherry and GFP), then following the individual cell back in time to see if hZAP-GFP-containing SGs ever formed in that cell. With respect to the infected hZAP-GFP cells, every infected cell (22/22 cells, 100%) showed ZAP-containing SGs at some point prior to or during active infection (Fig 4B and S1 Appendix and S1 Movie).

Next, to see if ZAP localization to SGs is always associated with virus infection, individual cells that showed hZAP-GFP punctae at any time at or before 18 hours into the 24-hour imaging period were tracked over the entire time course to see if nsP3-mCherry would accumulate. The 18-hour cutoff was chosen since many cells formed hZAP-GFP punctae that preceded nsP3-mCherry accumulation by 2–4 hours. Therefore, if cells formed punctae near the end of the imaging period it would be unclear if they would eventually show signs of virus replication, i.e. nsP3-mCherry signal. 6 hours was chosen as a conservative cutoff for a follow up period. Interestingly, the majority of cells that showed ZAP localization to SGs did not show any signs of active virus replication, i.e. no nsP3-mCherry signal (Fig 4C and S1 Movie), with only 41.5%, 22 out of 53 cells total, becoming infected at later timepoints (the same cells as those in previous paragraph) (S1 Appendix and S1 Movie). The trigger causing ZAP targeting to SGs was not obvious as many of the cells in the infected cultures showing localization of ZAP to SGs were in close proximity to infected cells, but not every cell adjacent to an infected cell showed this localization (Fig 4B and S1 Movie). Collectively, these data suggest that virus entry or early replication may be the necessary trigger for ZAP localization to SGs.

### ZAP localization to SGs likely requires virus infection and/or replication in the SG-forming cell

**Fig 5:**
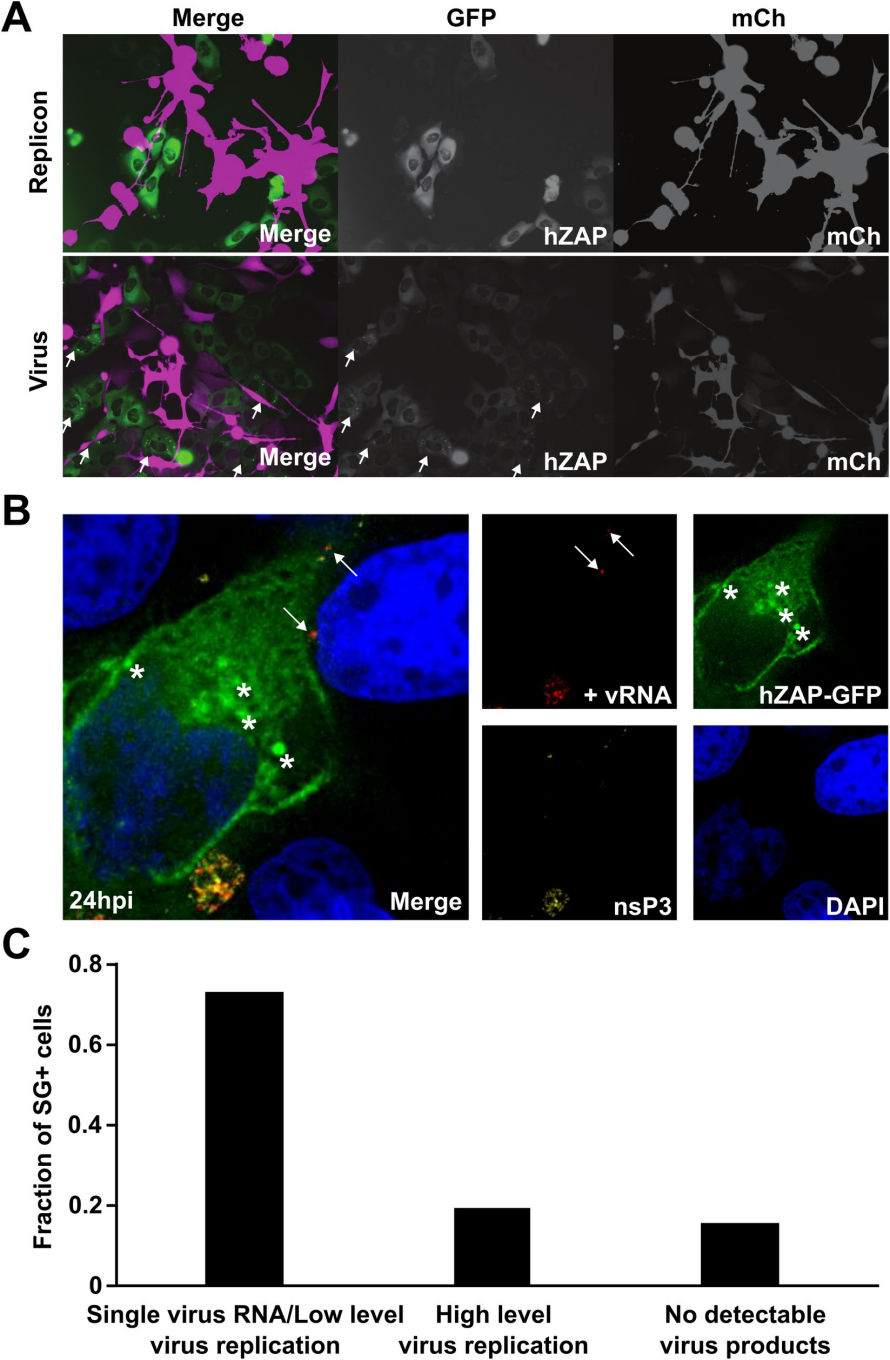
Virion entry is likely required for SG localization of ZAP. (A) U2OS cells stably expressing hZAP-GFP were co-cultured with BHK cells electroporated with RNA encoding either a SINV replicon expressing mCherry (top panels) or SINV genome expressing mCherry from a second subgenomic promoter (TE/5’2J/mCherry, lower panels). Localization of ZAP in the U2OS cells under these conditions was monitored by time-lapse imaging. Representative images at 36 hpi are shown. Arrows indicate cells in which ZAP has localized to SGs. (B) Naïve U2OS cells were cultured with U2OS cells expressing hZAP-GFP at a mixture of 4:1 and were infected with SINV expressing mCherry-tagged nsP3 (MOI = 1). After 24 hr, cells were fixed and analyzed by single-molecule fluorescent in situ hybridization (smFISH) using probes to detect the subgenomic region of the positive strand RNA (+vRNA) to track incoming infection and virus replication. Individual images are shown to the right and a merge image of a representative field is shown to the left. Asterisks indicate SG-localized ZAP in a cell with + vRNA (white arrows). (C) Images obtained of the fields in 5B were quantified for the number of cells demonstrating SG-localized ZAP (26 cells), the level of +vRNA, and presence of nsP3-mCherry in those cells. The fraction of SG-containing cells with high +vRNA and detectable nsP3-mCherry, single or low level +vRNA without nsP3-mCherry or no evidence of SINV infection is plotted. See also S2 Appendix.

Since ZAP localizes to SGs in infected cultures (Fig 1B), and SGs can form in cells without obvious SINV infection (Fig 4C and S1 Appendix and S1 Movie), we hypothesized that infected cells might send a signal causing SGs to form in other cells or that virus released from an infected cell might trigger the observed ZAP phenotype in another cell in the culture. In order to address these possibilities, we tested if ZAP localization to SGs occurs in cells co-cultured with either BHK cells harboring a SINV replicon or BHK cells infected with SINV. The replicon allows viral RNA replication and cytopathic effect to occur in the absence of virion production, thereby stressing the cells in a similar manner as virus infection but without virus production and spread. The infected BHK cells allow viral RNA replication and cytopathic effect to occur in the presence of virion production. Thus, if ZAP localization to SGs occurs in the presence of the replicon coculture, cells most likely produce a ‘stress’ signal that causes the localization event in neighboring cells. If virion production in the BHK cells is required the trigger is most likely virus entry and possibly replication. Co-culturing of U2OS cells with BHK cells harboring the SINV replicon did not cause ZAP relocation in the U2OS cells. In contrast, in U2OS cells co-cultured with virion-producing BHK cells, many examples of ZAP localization to SGs were evident (Fig 5A). Consistent with this result is the finding that IFN beta addition does not induce ZAP localization to SGs (S2 Movie). This suggests that virions produced by infected cells trigger ZAP localization to SGs in nearby cells.

It was unclear if both virion entry and subsequent virus replication are necessary for SG formation, or if virion entry and exposure to the genome was sufficient. To this end, UV-inactivated SINV was added to U2OS cells. Interestingly, infection with UV-inactivated SINV does not cause ZAP recruitment to SGs (S1 Fig) suggesting that viral replication processes are necessary. Additionally, poly (I:C) stimulation of U2OS cells is sufficient for ZAP recruitment to SGs (S2 Fig), but recombinant IFN beta addition is not (S2 Movie). Since genome RNA (UV-inactivated SINV) did not cause ZAP recruitment to SGs but the double strand RNA mimic poly(I:C) did, the data collectively suggest that cells sense a threshold level of dsRNA induced by virus replication processes, causing the formation of visible SGs and recruitment of ZAP.

Next to directly test if early viral events have taken place in cells in which ZAP was targeted to SGs, smFISH was performed to detect viral RNA in U2OS cells exposed to SINV (Fig 5B and 5C). smFISH tiles multiple fluorescently-tagged probes onto a single mRNA leading to a diffraction limited spot, allowing detection of single RNA molecules [52]. Using this technique, it is possible to find single-infection events and assess whether these are present in SG-containing cells. Cells were infected with SINV, then fixed, and stained at 0, 1, and 24 hpi. Single viral RNA molecules could be detected at all time points (S3 and S4 Figs). Based on the time-lapse microscopy experiments (Fig 4), the 24 hr time point was chosen to analyze how SG formation corresponds to the presence of viral RNA and the presence of detectable viral protein (nsP3-mCherry). At 24 hpi many cells showed SG formation. Strikingly, of the cells with detectable SGs, we found that the vast majority (>70%) contained detectable, but low levels of SINV RNA, without evidence of nsP3 accumulation (Fig 5B and 5C and S2 Appendix). This result is consistent with the scenario that the presence of viral RNA is sufficient to trigger SG formation and thus hZAP localization to SG punctae. The presence of low levels of SINV RNA as detected by smFISH (Fig 5) in most cells with SGs, in conjunction with the fact that many cells that exhibit SGs do not become infected over the course of 24 hr (Fig 4C and S1 Movie), suggest that hZAP relocation to SG formation may either: (*i*) clear virus products from infected cells, or (*ii*) halt virus replication for a certain period of time. Importantly, either scenario attenuates virus spread (Fig 1).

### Identification of the SG targeting signal of ZAP and its anti-SINV role

**Fig 6:**
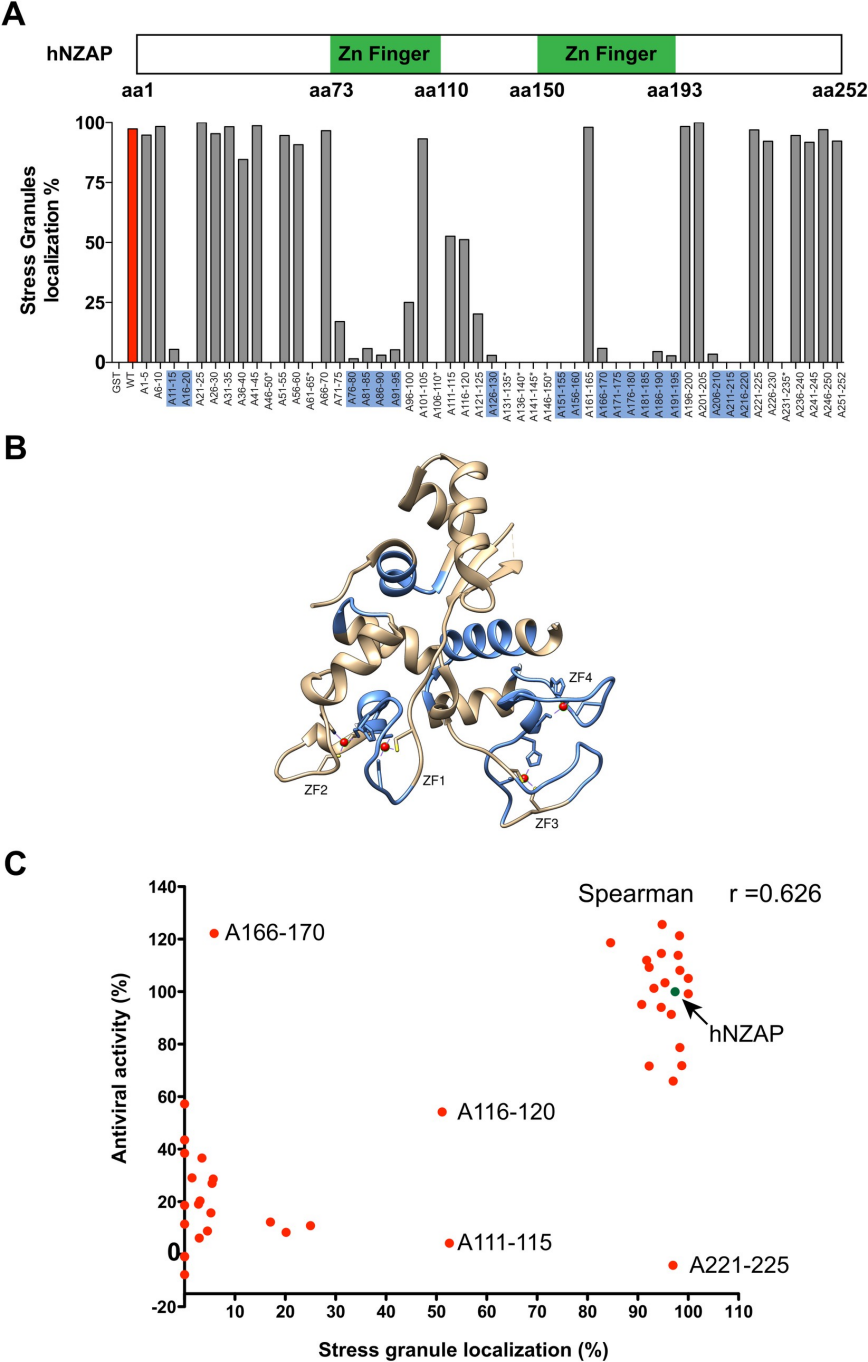
ZAP antiviral activity correlates with its ability to localize to SGs. (A) A previously described panel of 51 alanine mutants of GST-NZAP [18] in which blocks of 5 amino acids were replaced with 5 alanines within the minimal antiviral domain of ZAP (amino acids 1–252) were tested for SG localization under conditions of oxidative stress. A schematic of the antiviral domain of human ZAP (hNZAP) is shown at the top for reference. U2OS cells were transfected with the indicated mutants, with numbers indicating the amino acid residues replaced with alanine, and after 48 hr the cells were treated with 0.5 mM arsenite for 30 min and evaluated for ZAP and SG colocalization as in Fig 1. For each transfection, at least 50 transfected cells were assessed for ZAP localization in SGs as defined by colocalization with the SG marker TIA-1 and the percentage with SG localization is plotted. Results show an average of two independent transfections. Constructs exhibiting low expression either due to reduced stability or poor transfection are marked with an asterisk. Mutation of amino acids 11–20, 76–95, 126–130, 151–160, 166–195, and 206–220 (highlighted in blue) severely affected SG localization of ZAP (<10% of ZAP-transfected cells with SG localization). GST alone served as negative control. (B) Ribbon diagram of the rat NZAP structure. The SG-targeting residues in hZAP (highlighted in blue in Fig 6A) are highlighted in blue on the previously solved crystal structure of NZAP225 (PDB: 3U9G; [20]) using UCSF Chimera [62]. The cysteine and histidine side chains coordinated to zinc atoms (red) are shown in a ball-and-stick representation. ZF: zinc finger. (C) For each ZAP mutant, SG localization and anti-SINV activity, as measured by a flow cytometry-based assay and previously reported [18], are plotted. Correlation was assessed by GraphPad Prism 5 (Spearman R = 0.626). The arrow indicates results with wildtype hNZAP; selected mutants are indicated.

To map the amino acid regions of ZAP important for localization to SGs, we used a previously generated panel of NZAP mutants in which 5 amino acid blocks of residues within the antiviral domain of ZAP were replaced with alanines [18]. Cells expressing the various NZAP constructs were subjected to oxidative stress and SG localization was quantified by imaging. Mutations in several regions, such as amino acids 11–20, 71–100, 121–160, 166–195 and 206–220, were deleterious for SG targeting (Fig 6A). Most of these mutations severely affected ZAP localization to SG (<10% of ZAP-expressing cells, highlighted in blue in Fig 6A). When residues 111–115 or 116–120 were replaced with alanines, the mutants showed a partial reduction in SG targeting. There were 8 mutants with poor expression (indicated with asterisks in Fig 6A), and these residues could not be assessed for their effect on SG targeting. Collectively, these data show that multiple regions throughout the NZAP domain of ZAP are critical for SG targeting.

Fig 6B shows the location of the most critical SG-targeting residues (Fig 6A, highlighted in blue) mapped on the previously determined crystal structure of rat NZAP [20]. Of these regions important for SG targeting, amino acids 76–95 overlap with the first 2 zinc fingers of ZAP (referred to as ZFs in Fig 6B) while amino acids 151–160 and 166–195 regions overlap with the last 2 zinc fingers. These zinc fingers are critical for RNA binding and the antiviral activity of the protein [24].

Next, we examined the relationship between SG targeting and anti-SINV activities of ZAP, based on our previous assessment of the antiviral activity of each mutant [18]. As shown in Fig 6C, where the SG localization and antiviral activity for each mutant are plotted, the majority of the ZAP mutants were clustered either in the lower left quadrant (poorly antiviral with markedly reduced SG localization) or in the upper right quadrant (highly antiviral and able to localize to SGs). Thus, the anti-SINV activity of ZAP correlates with the ability to localize to SGs (Spearman coefficient of 0.626). There were a few exceptions that fell into 3 categories. The A116-120 ZAP mutant was intermediate in both its ability to inhibit and to localize to SGs, with SG localization of ZAP only seen in about half the cells. The A111-115 and A221-225 mutants were intermediate or normal in their ability to localize to SGs but were unable to inhibit SINV, suggesting SG targeting alone might not be sufficient for ZAP’s antiviral activity. In contrast, the A166-170 mutant retained antiviral activity but was unable to localize to SG under oxidative stress. However, the A166-170 mutant does localize to SG after SINV infection (S5 Fig). This mutant corroborates previous observations that SG composition is correlated with the activation signal [53]. Thus, the A166-170 mutant shows that ZAP can be differentially targeted to SGs under various stress conditions and that different portions of ZAP may be important for SG localization under different stress conditions. Taken all together the data suggest that different stressors can localize ZAP to SG and targeting to SGs is correlated with the antiviral activity of ZAP.

## Discussion

In this study, the molecular cues that lead to ZAP localization to SGs and the role of SG targeting in the anti-SINV function of ZAP were examined. Live-cell fluorescence imaging showed ZAP localization to SGs is a transient process (Fig 4C). The signal triggering the formation of ZAP-containing SGs requires virion infection (Fig 5) and virus replication within the infected cells (S1 Fig). Subsequent analysis by smFISH revealed the presence of SINV RNA in the majority of cells showing ZAP localization to SGs (Fig 5). Using a previously established panel of alanine mutants [18], we found that a multipartite SG targeting signal in ZAP exists that responds to cellular stress. Importantly, these regions have unique roles as one mutant only targets to SGs under a particular stress condition (Fig 6 versus S5 Fig). The prominent regions important for SG targeting overlap with the zinc-fingers important for RNA binding activity [24]; it is thus not surprising to find that SG targeting and antiviral function are correlated (Fig 6).

By following ZAP with live-cell imaging, we found that SG formation during SINV infection was highly dynamic with some SGs forming and dissolving (Fig 4B). In ZAP overexpressing cells, there were many examples of cells showing multiple SG formation and dissolution events, but the majority of these cells did not accumulate viral nsP3-mCherry over the imaging time course (S1 Appendix). In cells expressing tagged TIA-1, SINV infection triggered the formation of SGs followed by accumulation of viral protein and subsequent cytopathic effect (Fig 4A and S1 Appendix and S1 Movie), suggesting that SG formation itself is insufficient to block SINV replication. These results lead to a hypothesis that overexpression of ZAP could prevent productive SINV infection and thus allow the subsequent dissolution of the SINV triggered SGs. The recurrence of SGs in the same cell could indicate either multiple incidents of non-productive infection or multiple delays in the onset of a single infection event.

Collectively, these data show an interesting timeline in the dynamics of SINV and ZAP during virus infection. At early timepoints post infection, ZAP can localize to SGs, accumulating in a matter of minutes and dissolving in a matter of hours (Figs 1 and 4 and S1 Movie). The SG formation proceeds any visible nsP3-mCherry protein expression (Fig 4 and S1 Movie). The trigger for this stimulus can be a few viral RNA molecules (Fig 5). As many of these cells do not eventually produce nsP3-mCherry and continue to grow (Fig 4), it is clear that the infection process was halted, or perhaps these cells were infected with defective particles. Alternatively, ZAP, viral nsP3, and viral RNA can all colocalize in high amounts (Figs 4 and S4), and the live-cell microscopy shows that high-levels of nsP3-mCherry accumulation eventually leads to cell death (S1 Movie). Thus, ZAP localization can occur at various stages of virus infection and can lead to different phenotypic outcomes for a particular cell (Fig 4 and S1 Appendix and S1 Movie).

The co-culture experiments showing that only cells producing virions could trigger the formation of ZAP-containing SGs (Fig 5A) are consistent with the idea that incoming virions trigger the formation of SGs (Fig 5B). This is further supported by the results using the sensitive smFISH technique, where low levels of incoming viral RNA are detectable in cells showing SG localization of ZAP (Fig 5B). It is possible the ZAP-containing SGs could block translation of the SINV genome, preventing productive infection, with subsequent dissolution of the SGs. It has been reported that ZAP acts as a sensor of MLV RNA and that ZAP and viral RNA colocalize [54]. More recently, ZAP has been shown to bind to CG dinucleotide motifs in the HIV genome leading to inhibition of virion production [16].

Although ZAP’s targeting to SG and its anti-SINV activity are correlated (Fig 6C), some ZAP mutants that localize to SG are non-functional, indicating additional features of the ZAP protein are needed, possibly for recruitment of additional host factors. It has been shown ZAP synergizes with interferon inducible factors to exert its anti-SINV activity in BHK cells [27]. Furthermore, ZAP has been shown to interact with other host factors such as exosome components [21] and the innate immune sensor TRIM25 [26]. Further work is needed to determine if any of these factors work with ZAP to exert its anti-SINV activity via the SG-related pathway shown in this study. Of the most recently identified ZAP-interacting partners, TRIM25 has been shown to be targeted to SG during virus infection [55,56]. The ubiquitination and oligomerization of TRIM25 is required for its interaction with ZAP, but the cellular location of this interaction is not defined yet. Further characterization of the ZAP-TRIM25 interaction and the possible role of SGs could lead to a better understanding of their antiviral mechanism.

ZAP is a unique antiviral protein effective against diverse viruses. Although the underlying antiviral mechanism requires further characterization, ZAP targeting to SGs is important for its anti-SINV function. It would be of interest to test whether this is a general mechanism by which ZAP blocks other ZAP-sensitive viruses as this will help further illuminate the antiviral role of SGs. In addition, understanding the antiviral mechanism of ZAP could provide a road map to design pan-viral inhibitors to combat diverse viruses.

## Material and methods

### Cell lines

Cell lines were maintained at 37°C in a humidified atmosphere containing 5% CO_2_. BHK-J cells [57], a derivative of BHK-21 (ATCC CCL-10; hamster kidney fibroblasts), were cultured in minimum essential medium (Invitrogen, Carlsbad, CA) supplemented with 7.5% fetal bovine serum (FBS). 293T cells (ATCC CRL-11268), a derivative of human embryonic kidney 293 cells expressing the simian virus 40 T antigen and selected for adherence and transfectability, were generously provided by Guangxia Gao and Stephen P. Goff (Columbia University, New York, NY), and cultured in Dulbecco’s Modified Eagle medium (DMEM; Invitrogen) supplemented with 10% FBS. U2OS (ATCC HTxB-96; human osteosarcoma epithelial cells) were cultured in DMEM containing 10% FBS (complete media). For time-lapse imaging, cells were either maintained in CO_2_-independent media (Invitrogen) containing 10% FBS, 1 mM sodium pyruvate and 2 mM L-glutamine (imaging media) or normal growth medium. Recombinant interferon beta 1a (IFN-beta) (PBL Assay Sciences, Cat 11410–2) was added at 4070 units per mL, as measured by activity.

### Plasmid constructs

Constructs were created using standard molecular biology methods. Plasmid pToto1101/Luc has been previously described [10]. Various reporters and subcellular localization markers were constructed in a lentivirus backbone derived from TRIP-EGFP [58] or TRIP-RFP. TRIP-RFP was constructed by replacing the coding sequence of EGFP with the TagRFP sequence from pTagRFP-C (Evrogen). TRIP-EGFP-hZAP was constructed by inserting the PCR-generated coding sequence of hZAP [short isoform (Q7Z2W4-2) encoded by gene *ZC3HAV1*] into the BsrGI/XhoI sites of TRIP-EGFP (used to make the U2OS hZAP-GFP lines). TRIP-EGFP-TIA-1 was constructed similarly by inserting PCR-generated coding sequence of TIA-1 into BsrGI/XhoI sites of TRIP-EGFP. To construct TRIP-RFP-DCP1, the DCP1 coding sequence was amplified by PCR from plasmid mRFP-DCP1a [51] and then inserted into the BsrGI/XhoI sites of TRIP-TagRFP. GST-tagged hNZAP plasmid containing the minimal anti-SINV domain and mutants were described previously [18].

### Viruses

SINV expressing mCherry as a fusion with nsP3 (Toto1101/mCherry) or expressing blue fluorescent protein (BFP) as a fusion with nsP3 (Toto1101/BFP) were constructed by replacing the enhanced green fluorescent protein (EGFP) coding region of Toto1101/GFP [59] with the coding sequence of mCherry (derived from the TRIP-mCherry-CLDN1 plasmid [60] or BFP (Evrogen), respectively, using the flanking SpeI sites. SINV encoding EGFP from a duplicated subgenomic promoter (TE/5’2J/GFP) and a SINV replicon expressing mCherry from the subgenomic promoter were previously described [61]. SINV expressing mCherry from a duplicated subgenomic promoter was generated by replacing the GFP sequences in TE/5’2J/GFP with sequences encoding mCherry.

For preparation of virus stocks or expression of replicon RNA, in vitro transcribed SINV genomic RNA was electroporated into BHK cells as described [10]. Virus titers were determined on BHK-J cells using 10-fold serial dilutions of sample and visualization of plaques with crystal violet staining, also as previously described [10]. Multiplicities of infection (MOI) were calculated based on these BHK- J-derived titers. The relative infection frequencies between BHK and U2OS cells were measured as well (S6 Fig). UV-inactivated SINV was produced by exposing virus in a thin film of liquid to 254nm light at 9999uJxcm^2^ for 10 minutes.

### Generation of lentivirus pseudoparticles and transductions

Pseudoparticles (pp) were generated by co-transfection of 293T cells with TRIP provirus-, HIV gag-pol-, and vesicular stomatitis virus envelope protein G (VSV-G)-expressing plasmids using a weight ratio of 1: 0.8: 0.2, as described previously [60]. U2OS cells were transduced by incubation for 6 hr at 37°C with TRIPpp diluted 1:3 or greater to prevent cytotoxicity in complete media supplemented with 4 μg/ml polybrene and 20 mM HEPES. In some cases, transduced cell populations were enriched for greater expression using a FACSAria II high-speed flow cytometry cell sorter (BD Biosciences).

### Transfection of U2OS cells

U2OS hZAP-GFP cells were transfected with poly (I:C) at 1ug/mL. Briefly, poly (I:C) was incubated with X-tremeGENE^TM^ (Sigma-Aldrich) at a 1:4 ratio in Opti-MEM (Thermo Fisher Scientific) and incubated at room temperature for 15 minutes. The mixture was then added dropwise to cells before live-cell microscopy.

### Fixed-cell microscopy

Cells were fixed using 3.7% formaldehyde and permeabilized using saponin. GST, TIA-1 and 4E-T were detected using anti-GST (26H1, Cell Signaling Technology), anti-TIA-1 (C-20, Santa Cruz) and anti-4ET (sc-514810, Santa Cruz) respectively, followed by secondary antibodies conjugated with Alexa fluor dyes (Invitrogen). Confocal imaging of fixed samples was performed using an inverted Axiovert 200 laser scanning microscope (Zeiss).

For the single-molecule RNA fluorescence in situ hybridization, U2OS naïve and U2OS hZAP-GFP cells were mixed at a 4:1 ratio and put onto 8-well chamber slides with #1.5 glass (Lab-Tek II). Cells were seeded at a confluency of 40%. The next day the media was aspirated and cells were infected in a minimal volume of PBS + 1% FCS with Toto1101/mCherry at a low MOI, and rocked for 1hr at 4 degrees C. The infection media was aspirated, normal growth media was added to the culture and cells were placed back into cell culture incubators. Cells were then fixed at 0, 1, and 24 hrs post infection (hpi) in 3.7% formaldehyde followed by 70% ethanol permeabilization at -20 degrees C overnight. The protocol for smFISH from Stellaris was followed. The probe set used for Sindbis smFISH can be found in the supplemental information. Imaging was performed on an inverted Olympus IX-70 microscope in a 60x oil 1.42NA objective with an Insight SSI 7 color solid state illumination system. DAPI, GFP, AlexaFluor594, and AlexaFluor647 filter sets were used to image cell nuclei, hZAP-GFP, nsP3-mCherry, and smFISH probe sets. Images were deconvoluted and analyzed in ImageJ.

### Live-cell microscopy

For long-term live cell imaging, cells were grown on 1.5 Lab-Tek II 4-chambered coverslips (Thermo Fisher Scientific). Live cells maintained at 37°C in imaging media were imaged using a Zeiss Axiovert 200 inverted microscope equipped with an UltraView spinning disk confocal head (Perkin-Elmer), an Orca ER-cooled CCD camera (Hamamatsu), a 20×/0.75 N.A. Plan-Apochromat objective, and an environmental chamber (Solent Scientific). Solid-state 491 and 561 nm lasers (Spectral Applied) and ET 530/50 and ET 605/70 emission filters (Chroma) were used for excitation and emission of EGFP and RFP fluorescence, respectively. Alternatively, time-lapse images were captured using an Olympus IX71 inverted microscope equipped with an Orca ER cooled CCD camera, a 20×/0.75 N.A. UPlan SApo objective and an environmental chamber. Image acquisition was performed using Metamorph (Molecular Devices) and processing was performed using ImageJ.

## Supporting information

S1 Appendix Summary of results from SINV infection of U2OS Naïve and hZAP-GFP co-cultures (S1 Movie). Individual cells were tracked based on whether they were infected or formed SGs. The resulting infection phenotype was quantified on a per cell basis.
(XLSX)

S2 Appendix Summary of results from smFISH. The smFISH results from 32 individual fields examined at 24 hpi as described in Figs 4 and S4. Each position was first analyzed for ZAP localization, then for smFISH and nsP3 signals.
(XLSX)

S1 Movie Time-lapse microscopy imaging of SINV-infected U2OS Naïve and hZAP-GFP cells. Naïve U2OS cells were cultured with U2OS cells expressing hZAP-GFP at a mixture of 4:1 and were infected with SINV expressing nsP3-mCherry (MOI = 1). Images were taken every 20 minutes in the mCherry and GFP channel for 24 hours.
(AVI)

S2 Movie Time-lapse microscopy imaging of IFN-beta addition to U2OS hZAP-GFP cells. Recombinant IFN-beta was added to U2OS cells expressing hZAP-GFP. Images were taken every 30 minutes in the GFP channel for 15 hours.
(AVI)

S1 Fig UV-inactivated SINV does not induce SG localization of hZAP-GFP. U2OS hZAP-GFP or U2OS TIA-1 GFP cells were exposed to UV-inactivated or replication competent SINV. At 7 hrs post exposure, cells were imaged for GFP localization. Cells exhibiting punctae, a proxy for SG formation, are highlighted by the red arrows.
(TIF)

S2 Fig Poly (I:C) stimulation leads to hZAP-GFP localization to punctae. U2OS hZAP-GFP cells were transfected with Poly (I:C) and imaged by live-cell microscopy. Images were taken every 20 minutes for 15 hours. A cell exhibiting ZAP-containing SG punctae that then rapidly dissolve is highlighted by the white arrow.
(TIF)

S3 Fig Single-molecule FISH is able to detect individual virus RNA molecules at 0 and 1 hpi. Naïve U2OS cells were cultured with U2OS cells expressing hZAP-GFP at a mixture of 4:1 and were infected with SINV expressing nsP3-mCherry (MOI = 1). Cells were fixed and analyzed by smFISH using probes to the subgenomic region of the positive strand RNA (+vRNA) either immediately after infection (A) or after 1 hr (B). A merge image shows ZAP (GFP) in green, DAPI in blue, +vRNA in red (white arrows) and nsP3-mCherry (mCherry) in yellow. There was no observable nsP3 expression at either time point, as opposed to the image in Fig 4B taken at a later time point after infection. Data was obtained as described in the Materials and Methods.
(TIF)

S4 Fig SINV nsP3, RNA, and cellular ZAP colocalize in infected cells at 24 hpi. Naïve U2OS cells were cultured with U2OS cells expressing hZAP-GFP at a mixture of 4:1 and were infected with SINV expressing nsP3-mCherry (MOI = 1). Cells were fixed and analyzed by smFISH using probes to the subgenomic region of the positive strand RNA (+vRNA) after 24 hr. White arrows highlight areas of colocalization of hZAP-GFP, nsP3-mCherry and SINV RNA. Two z-slices from the same field of view, slice 32 and 4, are shown in (A) and (B), respectively. Data was obtained as described in the Materials and Methods.
(TIF)

S5 Fig Different regions of ZAP are important for localization to SGs depending on the stress signal. WT and the alanine A166-170 ZAP mutant each fused to GFP were overexpressed in U2OS cells as described in the Materials and Methods. Cells were exposed to SINV expressing nsP3-mCherry and examined 24 hr later by fluorescence microscopy. A representative field is shown; hZAP-GFP signal is in green and the SINV nsP3-mCherry is in magenta (image saturation occurs in white). Examples of SG localization of WT and the A166-170 mutant are highlighted by arrows.
(TIF)

S6 Fig U2OS cells are more refractory to SINV infection then BHK cells. BHK and U2OS cells were infected with SINV nsP3-mCherry at an MOI of 0.3, 3, and 30. The percent infected (mCherry positive) was measured by flow cytometry at 6, 12, and 24hpi.
(TIF)
