# Supplementary figures and images for: ZAP’s stress granule localization is correlated with its antiviral activity and induced by virus replication
Source: PLoS Pathog. 2019 May 22;15(5):e1007798. doi: 10.1371/journal.ppat.1007798 (PMC6548403; doi:10.1371/journal.ppat.1007798)

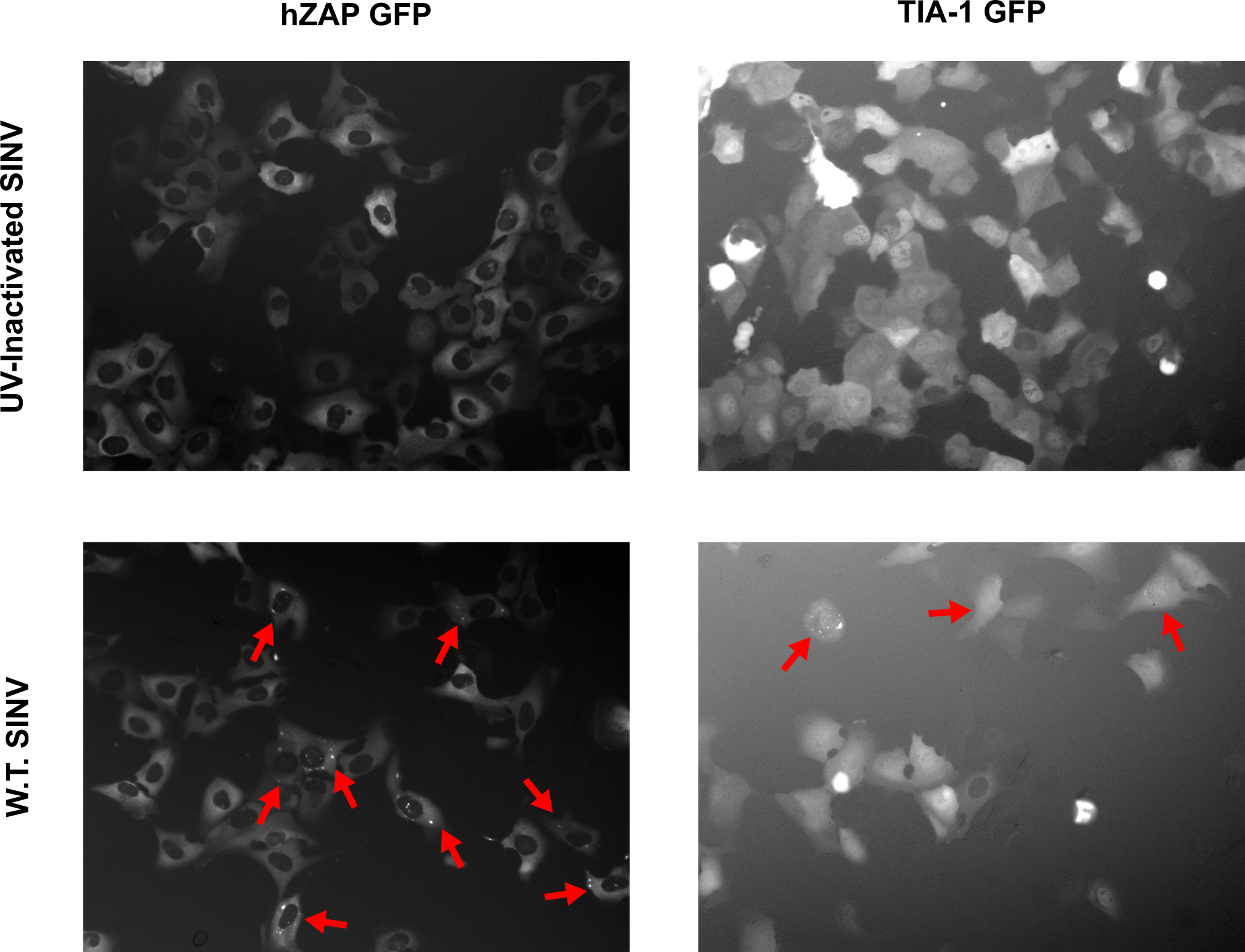

Supplement: S1 Fig — U2OS hZAP-GFP or U2OS TIA-1 GFP cells were exposed to UV-inactivated or replication competent SINV. At 7 hrs post exposure, cells were imaged for GFP localization. Cells exhibiting punctae, a proxy for SG formation, are highlighted by the red arrows. (TIF) [file ppat.1007798.s005.tif]

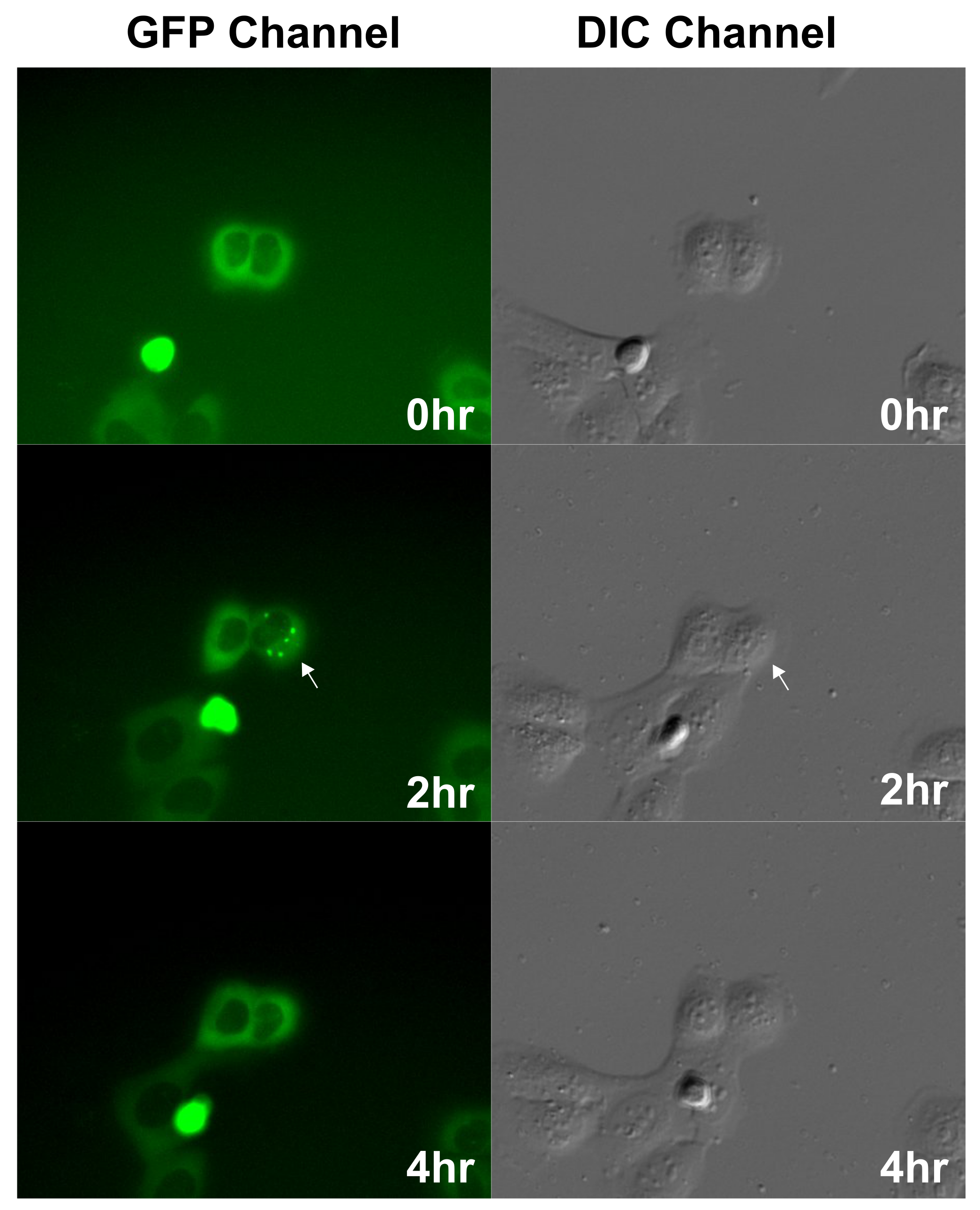

Supplement: S2 Fig — U2OS hZAP-GFP cells were transfected with Poly (I:C) and imaged by live-cell microscopy. Images were taken every 20 minutes for 15 hours. A cell exhibiting ZAP-containing SG punctae that then rapidly dissolve is highlighted by the white arrow. (TIF) [file ppat.1007798.s006.tif]

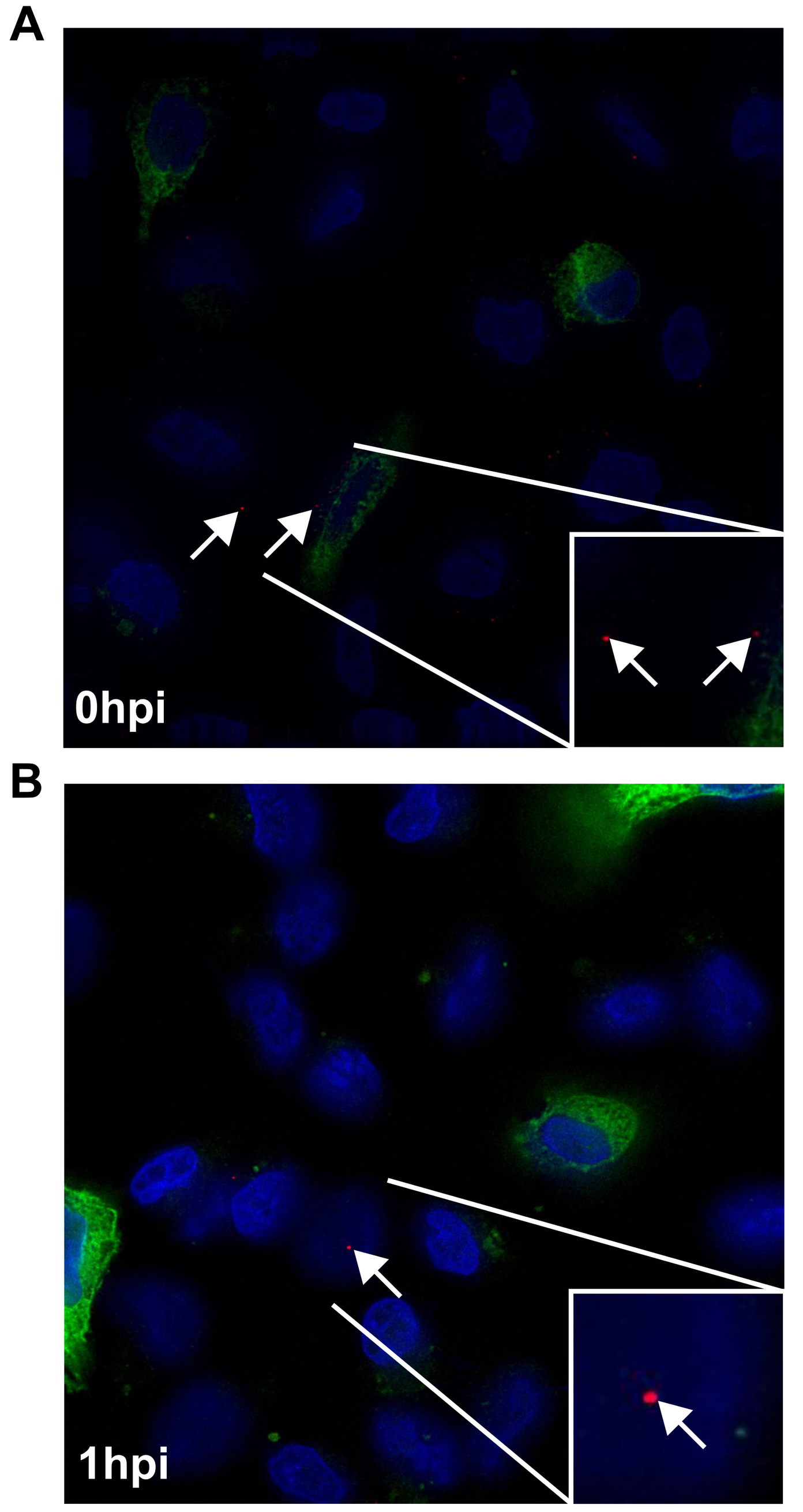

Supplement: S3 Fig — Naïve U2OS cells were cultured with U2OS cells expressing hZAP-GFP at a mixture of 4:1 and were infected with SINV expressing nsP3-mCherry (MOI = 1). Cells were fixed and analyzed by smFISH using probes to the subgenomic region of the positive strand RNA (+vRNA) either immediately after infection (A) or after 1 hr (B). A merge image shows ZAP (GFP) in green, DAPI in blue, +vRNA in red (white arrows) and nsP3-mCherry (mCherry) in yellow. There was no observable nsP3 expression at either time point, as opposed to the image in Fig 4B taken at a later time point after infection. Data was obtained as described in the Materials and Methods. (TIF) [file ppat.1007798.s007.tif]

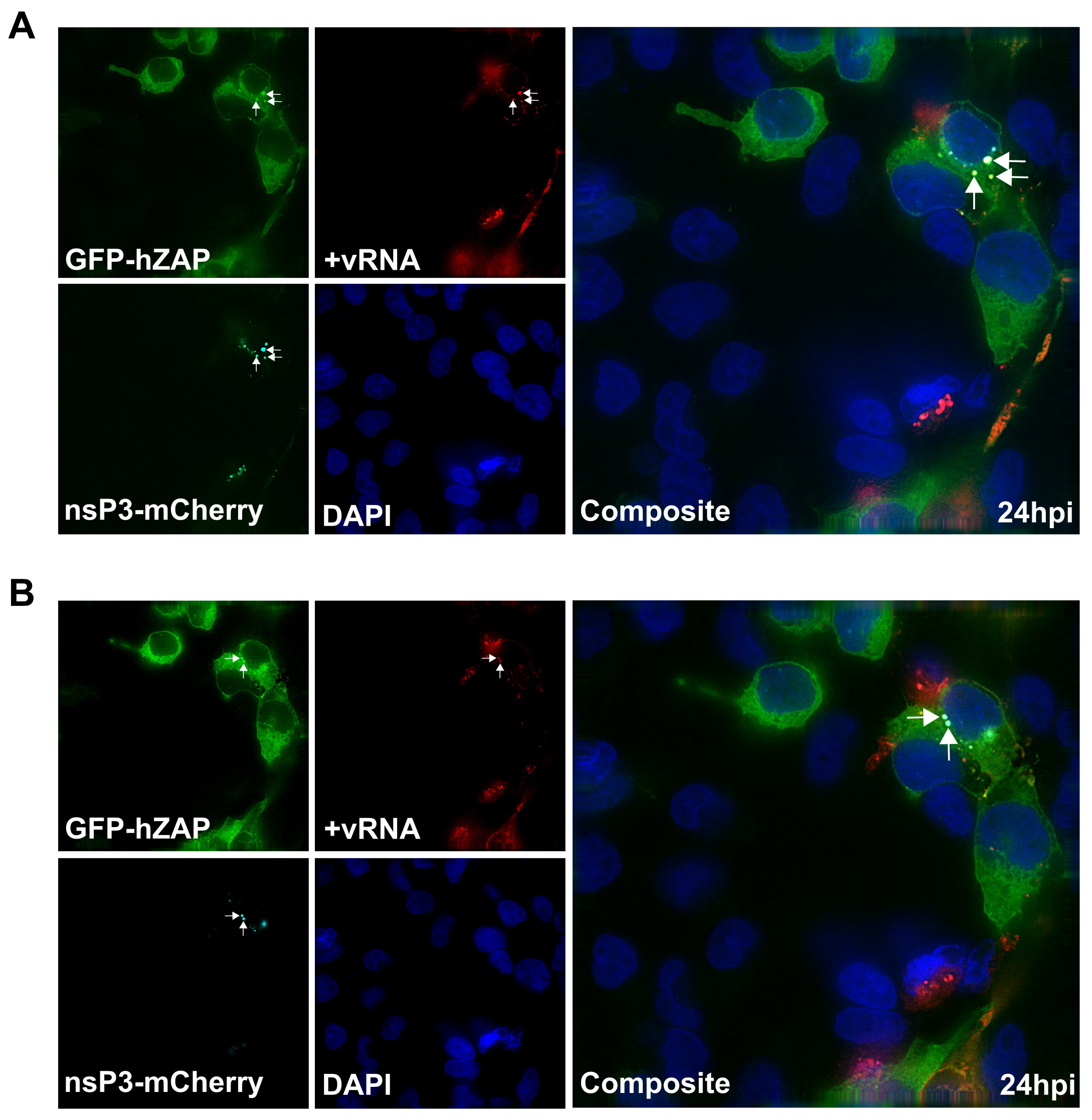

Supplement: S4 Fig — Naïve U2OS cells were cultured with U2OS cells expressing hZAP-GFP at a mixture of 4:1 and were infected with SINV expressing nsP3-mCherry (MOI = 1). Cells were fixed and analyzed by smFISH using probes to the subgenomic region of the positive strand RNA (+vRNA) after 24 hr. White arrows highlight areas of colocalization of hZAP-GFP, nsP3-mCherry and SINV RNA. Two z-slices from the same field of view, slice 32 and 4, are shown in (A) and (B), respectively. Data was obtained as described in the Materials and Methods. (TIF) [file ppat.1007798.s008.tif]

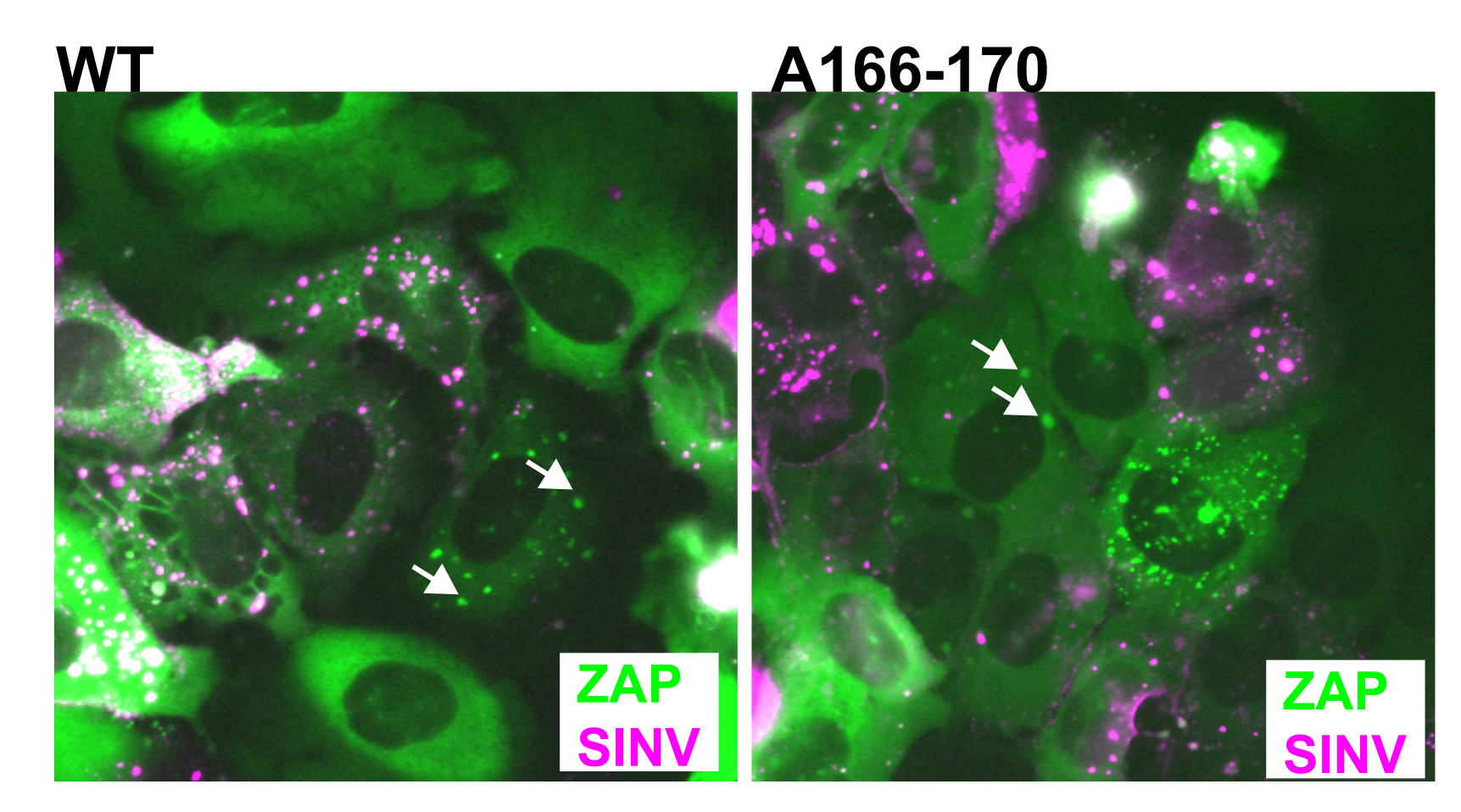

Supplement: S5 Fig — WT and the alanine A166-170 ZAP mutant each fused to GFP were overexpressed in U2OS cells as described in the Materials and Methods. Cells were exposed to SINV expressing nsP3-mCherry and examined 24 hr later by fluorescence microscopy. A representative field is shown; hZAP-GFP signal is in green and the SINV nsP3-mCherry is in magenta (image saturation occurs in white). Examples of SG localization of WT and the A166-170 mutant are highlighted by arrows. (TIF) [file ppat.1007798.s009.tif]

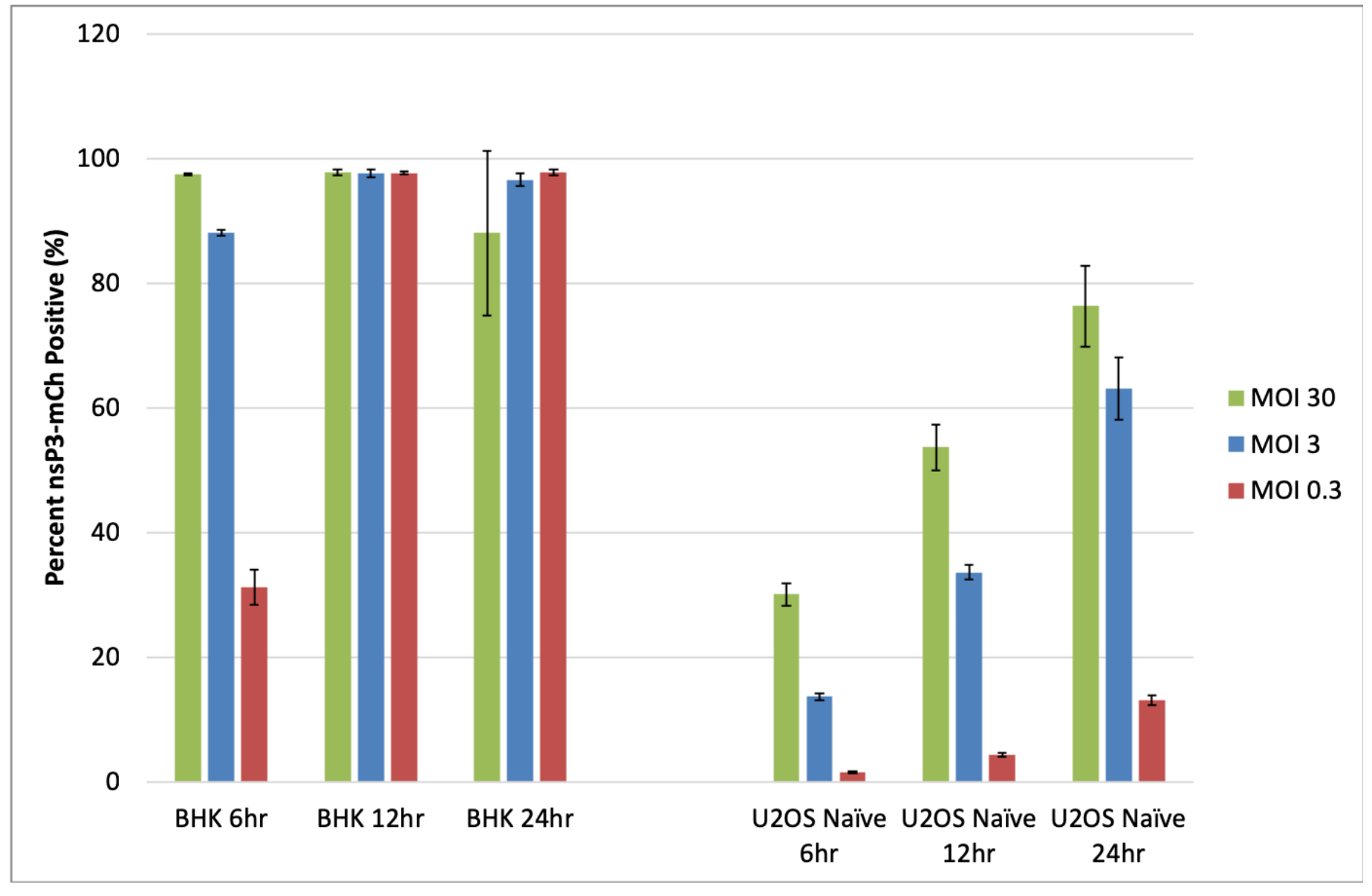

Supplement: S6 Fig — BHK and U2OS cells were infected with SINV nsP3-mCherry at an MOI of 0.3, 3, and 30. The percent infected (mCherry positive) was measured by flow cytometry at 6, 12, and 24hpi. (TIF) [file ppat.1007798.s010.tif]
